# Impact of novel Oral Poliovirus Type 2 vaccination campaigns: A seroprevalence survey in Nigeria, 2022

**DOI:** 10.1016/j.vaccine.2025.126978

**Published:** 2025-04-30

**Authors:** Zubairu Iliyasu, Abba Ahmed Danzomo, Visalakshi Jeyaseelan, Mustapha Modu Gofama, Patricia Eyanya Akintan, Olugbenga Oyewumi Akinrinoye, Umar Also, Hamisu Abdullahi, Kabir Yusuf Mawashi, Auwal Umar Gajida, Giovanna Sifontes, Bernardo A. Mainou, Rocio Lopez Cavestany, Ondrej Mach, Harish Verma

**Affiliations:** a Aminu Kano Teaching Hospital, Kano, Nigeria; b McKing Consulting Corporation, GA, USA; c Polio Eradication,WHO HQ, Geneva, Switzerland; d University of Maiduguri Teaching Hospital, Maiduguri, Nigeria; e Lagos University Teaching Hospital, Lagos, Nigeria; f University College Hospital, Ibadan, Nigeria; g Rasheeed Shekoni Specialist Hospital, Dutse, Nigeria; h WHO AFRO, Brazzaville, the Democratic Republic of the Congo; i National Primary Health Care Develoment Agency, Abuja, Nigeria; j US Centers for Disease Control and Prevention, Atlanta, GA, USA

## 1 Background

The Global Polio Eradication Initiative (GPEI) has made remarkable progress in reducing the global burden of wild poliovirus (WPV) transmission by >99 % since its inception in 1988. Currently, only two countries, Afghanistan and Pakistan, remain endemic for type 1 WPV (WPV1) [1]. In 2020, the African continent achieved the milestone of being certified WPV-free, with Nigeria reporting its last case of WPV1 in 2016 [2]. However, recent WPV1 cases in Mozambique and Malawi, genetically linked to strains detected in Pakistan, highlight the ongoing challenges in maintaining a polio-free status [3,4].

Polio eradication efforts are further complicated due to the spread of circulating vaccine-derived poliovirus (cVDPV). Sabin oral poliovirus vaccine (OPV) strains can, in rare circumstances, lose their attenuating mutations and generate VDPVs that are transmissible and neurovirulent. As such, poliovirus transmission, both of WPV and cVDPVs, officially recognized as a Public Health Emergency of International Concern (PHEIC), continues to stay.

To minimize the risk of serotype 2 vaccine associated paralytic poliomyelitis (VAPP) and cVDPV2, more importantly after the certification of global WPV2 eradication [5], trivalent OPV (tOPV, containing all 3 poliovirus strains) was withdrawn from routine immunization (RI) schedules in 2016 with a global switch from tOPV to bivalent OPV (containing types 1 and 3) [6]. At least one dose of inactivated poliovirus vaccine (IPV) was recommended to be introduced in RI to mitigate the resultant type 2 immunity gap. Nigeria introduced one dose of IPV (IPV1) into RI in 2015 at 6 weeks of age, and a second dose of IPV (IPV2) was added in 2021 at 14 weeks of age. WHO and UNICEF estimates of national immunization coverage report persistently sub-optimal IPV1 coverage at a sustained 62 % from 2020 to 2022 [8]; IPV2 coverage data is estimated to be 17 % and 37 % in the years 2021–2022 [7].

Since the 2016 switch, monovalent OPV type 2 (mOPV2) has been administered from a global stockpile to address outbreaks of cVDPV2. Nevertheless, the administration of mOPV2 has resulted in further seeding of cVDPV2 outbreaks in certain settings and conditions [9]. In order to address this issue, a modified version of mOPV2 was developed with enhanced genetic stability – called novel type 2 OPV (nOPV2) [10]. The modifications in nOPV2 aimed to reduce the risk of reversion to a neurovirulent phenotype while still maintaining comparable immunogenicity to mOPV2 for type 2 polioviruses. nOPV2 was clinically tested in randomized controlled trials and found to be safe and highly immunogenic [11,12]. In November 2020, nOPV2 was recommended by WHO prequalification for use in cVDPV2 outbreak response under Emergency Use Listing (EUL). In December 2023, nOPV2 received WHO prequalification, making it the first vaccine to reach full licensure after having been granted an EUL [13]. Since rollout began, nearly 1 billion doses of nOPV2 have been administered across 35 countries throughout the EUL period. Field data have allowed to estimate an 82 % reduction in the risk of cVDPV2 emergence with nOPV2 use compared to Sabin mOPV2 [14].

Nigeria has been facing ongoing transmission of cVDPV2 due to low population immunity and a large population with significant mobility. There were 16 paralytic cases and 5 environmental surveillance positive (ES+) isolates of cVDPV2 in 2020, then significantly increasing to 419 paralytic cases and 303 ES+ isolates in 2021 (WHO internal Polio Information Systems). In March 2021, Nigeria became the first country to use nOPV2 in response to a cVDPV2 outbreak. After the use of nOPV2, the number of cVDPV2 cases decreased to 48 AFP and 82 ES+ in 2022, and 85 AFP and 80 ES+ in 2023 (WHO internal Polio Information Systems). This reduction could potentially be attributable to the combined effect of the two IPV doses in RI and the use of nOPV2 in outbreak response. Nonetheless, cVDPV2 transmission persists despite multiple nOPV2 campaigns, setting the scene for the implementation of a seroprevalence survey to assess type 2 population immunity.

Seroprevalence surveys play a vital role in measuring population immunity, guiding immunization strategies, and assessing program performance in the context of polio eradication efforts [[15], [16], [17], [18]]. This seroprevalence survey after nOPV2 introduction was part of a series of serosurveys conducted in Nigeria over the years to compare poliovirus-specific immunity levels in different high-risk populations.

There were three main objectives in this seroprevalence survey: (1) to measure neutralizing antibody titers and seroprevalence to the three poliovirus serotypes, with a focus on type 2 poliovirus, (2) to explore the relationship between the number of nOPV2 doses / campaigns and type 2 seroprevalence, and (3) to examine any association between sociodemographic factors and type 2 seroprevalence.

## 2 Methods

### 2.1 Design and setting

**Fig. 1:**
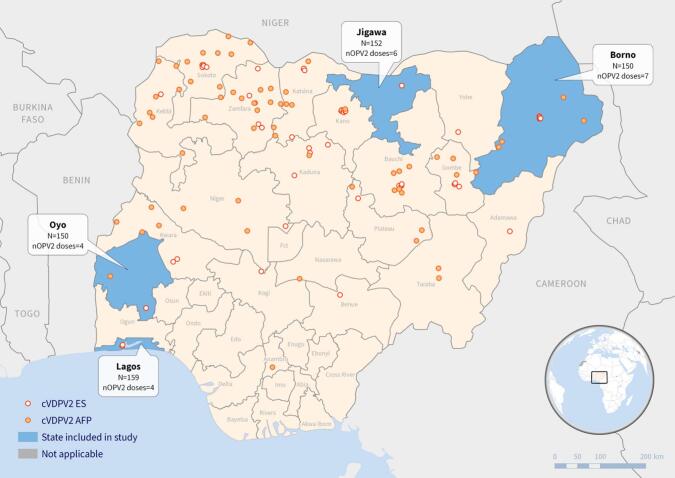
Map of Nigeria highlighting the states included in this nOPV2 seroprevalence study: Borno, Jigawa, Lagos and Oyo. The number of participants enrolled and number of nOPV2 campaigns from March 2021–November 2022 from each state are included. The positive environmental isolates and clinical acute flaccid paralysis cases in Nigeria are depicted in the year prior to study implementation (November 2021–November 2022). (Source: WHO Geographic Information Systems Centre for Health).

This cross-sectional, facility-based serosurvey was conducted in four states of Nigeria, selected based on specific epidemiological criteria, shown in Fig. 1. This study was implemented between October and November 2022. The states included were Borno due to experiencing the highest number of cVDPV2 cases among states with chronic security threats, Jigawa due to reporting the highest number of cVDPV2 cases overall, Lagos due to having a high population density, frequent population influx, and previous outbreaks of Ebola and COVID-19, and finally Oyo as a southern state that exhibited a significant number of cVDPV2 cases.

The largest public hospital with a high turnover of children within each of the four states were selected as study sites. These included the pediatric outpatient clinics at: the University of Maiduguri Teaching Hospital (Borno state), Rasheed Shekoni Specialist Hospital in Dutse (Jigawa state), Lagos University Teaching Hospital (Lagos state), and University College Hospital in Ibadan (Oyo state).

### 2.2 Study population

The study included children aged 6–59 months attending pediatric outpatient clinics for minor illnesses in the selected health facilities. These study sites are mostly accessed by patients from the neighboring catchment areas and few by referral from the peripheral sites. Eligible participants were children within the targeted age range (6–59 months), residing in the state for at least one month and whose parents/caregivers provided informed consent for participation. The study excluded acutely ill children requiring hospitalization, those with a history of bleeding disorders or contraindication to venipuncture, and those diagnosed or suspected to have a congenital immunodeficiency disorder.

### 2.3 Sample size determination

With an assumed type 2 seroprevalence of 76 % [19], a 95 % confidence interval, a design effect of 2, and a precision of 5 %, the minimum calculated sample size was 116 in each state. Accounting for a potential withdrawal rate of 20 % and insufficient blood samples, the sample size of 145 was increased and rounded up to 150 (a total of 600 children from all 4 states). To ensure an adequate representation of children aged ≤1 year, the participants were equally distributed into three age sub-groups: 6–12 months, 13–36 months, and 37–59 months. Each site was advised to enroll approximately 50 children in each age category, enabling comparison of seroprevalence and vaccination history across the different age groups.

### 2.4 Study procedures

During October–November 2022, parents/caregivers visiting health facilities with children aged between 6 and 59 months for minor ailments were informed about the survey by a study nurse in the pediatrics outpatient department (OPD). Parents who agreed to participate were directed to the study clinic after the OPD consultation. In the study clinic, the study physician provided comprehensive details about the study. Informed consent was obtained from parents willing to enroll their child in the survey. Children deemed “healthy” by the study physician, except for minor ailments, were confirmed for eligibility and the exclusion criteria. The study physician administered a questionnaire, which included socio-economic status of the parents and the immunization history of the child. The child's weight, height/length, and temperature were also measured.

### 2.5 Immunization history (Routine and Supplemental Immunization Activities (SIA) doses)

Immunization history was important to link the seroprevalence outcome with number of polio vaccine doses presumably received by each participant. An elaborate training was provided to the study physicians on the prevailing immunization schedule in the country and number of SIAs conducted in their respective catchment areas.

The RI schedule for polio vaccinations in Nigeria includes four doses of bOPV (at birth, 6, 10, and 14 weeks) and two doses of IPV (at 6 and 14 weeks of age).

From 2021 to 2022, Nigeria implemented four to eight SIAs, known as immunization plus days (IPDs), using nOPV2 in different states for outbreak response to cVDPV2. The number of doses reported should normally match the number of nOPV2 campaigns implemented in that population, but it does not happen in reality because of variable quality of coverage. The number of nOPV2 doses expected to have been received by children in the four states from IPDs in 2021–2022 were as follows: Borno (7 doses), Jigawa (6 doses), Lagos (4 doses), and Oyo (4 doses).

In addition, Borno and Jigawa conducted a single IPV campaign each in 2018 and Borno carried out an additional fIPV campaign in 2019. During IPDs, all children up to 5 years are expected to receive an additional vaccine dose, regardless of their previous immunization status.

The number of IPDs with different polio vaccines (nOPV2, mOPV2, IPV/fIPV and bOPV) in the 5 years preceding the survey were made available to the research physicians for reference during history taking. Immunization history was based on caregiver recall and immunization cards when available.

### 2.6 Blood sample collection

A trained phlebotomist drew 1–2 ml of peripheral blood from eligible participants in a vacutainer with all necessary precautions. The collected blood samples were then centrifuged in the laboratory at respective health facility. The extracted sera were stored in a deep freezer and later shipped to the Centers for Disease Control and Prevention (CDC) in Atlanta, USA, in dry ice maintaining a temperature of −20 °C or below. The assay used Sabin strains for all three poliovirus serotypes, following standard microneutralization protocols used by the CDC [20].

### 2.7 Data entry and analysis

The outcome of interest was seroprevalence; seropositivity is defined as children with detectable antibody levels at a dilution of 1: ≥8. Seroprevalence was expressed as a percentage with Clopper-Pearson 95 % confidence intervals. Median titers with Bootstrap 95 % confidence intervals were also done. Bivariate associations between seroprevalence and selected socio-demographic variables, anthropometric indices and immunization history were assessed. Additionally, adjusted analysis using generalized linear model with logit transformation was conducted for type 2 seropositivity with all risk factors that were considered relevant.

### 2.8 Ethical considerations

Ethical approval was obtained from each participating health facility, the National Health Research Ethics Committee, and the WHO Ethics Review Committee. The study procedures adhered to the principles of good clinical practices. This activity was reviewed by CDC and was conducted consistent with applicable federal law and CDC policy (see e.g., 45C.F.R. part 46, 21C.F.R. part 56; 42 U.S.C. §241(d); 5 U.S.C. §552a; 44 U.S.C. §3501 et seq.).

## 3 Results

**Fig. 2:**
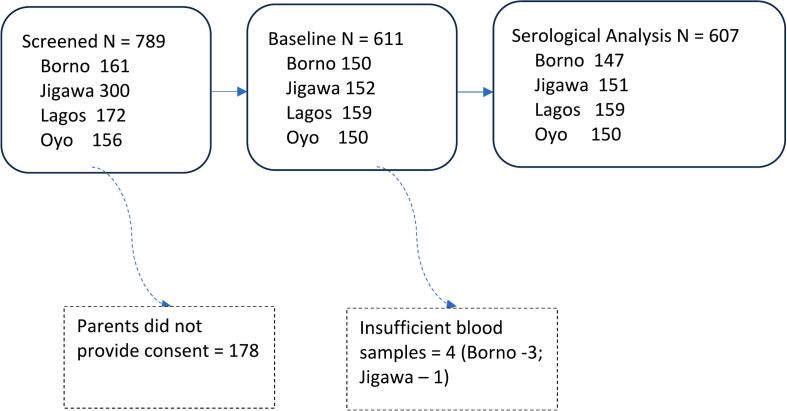
Consort table.

A total of 789 eligible children were screened of whom 178 parents did not provide consent for their child's participation in the survey. Ultimately, 611/789 (77.4 %) children were enrolled at the four sites, and 607/611 (99.3 %) children who provided adequate serum samples constituted the analyzable sample (Fig. 2).

Among the enrolled children, approximately one-third belonged to each of the three age categories [6–12 months (32.8 %; 201/611), 13–36 months (33.9 %; 207/611) and 37–59 months (33.2 %; 203/611)] combined the four study sites. Of the total children, 43 % (262/611) were female. Regarding parental education, 12.5 % (76/611) of the mothers and 7.8 % (47/611) of the fathers of the enrolled children had no formal education (Table 1).

**Table 1:** Baseline distribution of socio-demographic and anthropometric characteristics in the study sample, by age group and overall.

Baseline characteristics	Age groups						Total (N=611)	
	6–12 months (*N* = 201)		13–36 months (*N* = 207)		37–59 months (*N* = 203)			
	n	%	n	%	n	%	n	%
Female Children	98	48.8	85	41.1	79	38.9	262	42.9
Sites								
Borno	50	24.9	50	24.2	50	24.6	150	24.5
Jigawa	50	24.9	52	25.1	50	24.6	152	24.9
Lagos	51	25.4	55	26.6	53	26.1	159	26.0
Oyo	50	24.9	50	24.2	50	24.6	150	24.5
Mother's education level								
Never attended School/No formal education	23	11.5	22	10.7	31	15.4	76	12.5
Primary/Secondary	68	34.0	78	37.9	74	36.6	220	36.2
Tertiary/Above	109	54.5	106	51.5	97	48.0	312	51.3
Father's education level								
Never attended School/No formal education	17	8.5	12	5.9	18	8.9	47	7.8
Primary/Secondary	51	25.6	55	26.8	50	24.8	156	25.7
Tertiary/Above	131	65.8	138	67.3	134	66.3	403	66.5
Number of children less than 5 years of age in household								
1	101	50.5	92	44.7	92	45.3	285	46.8
2	79	39.5	89	43.2	91	44.8	259	42.5
≥3	20	10.0	25	12.1	20	9.9	65	10.7
Underweight (weight-for-age Z score < −2)	54	26.9	55	26.6	34	16.8	143	23.4
Wasting (weight-for-height Z score < −2)	46	22.9	40	19.6	32	16.2	118	19.6
Stunting (height-for-age Z score < −2)	50	24.9	57	27.8	46	23.0	153	25.2

In terms of nutritional status, nearly one in four children (23.4 %, 143/611) were underweight, one in five children were wasted (19.6 %, 118/611), and one in four children (25.2 %, 153/611) were stunted (Table 1) among the enrolled children.

### 3.1 Seroprevalence

**Fig. 3:**
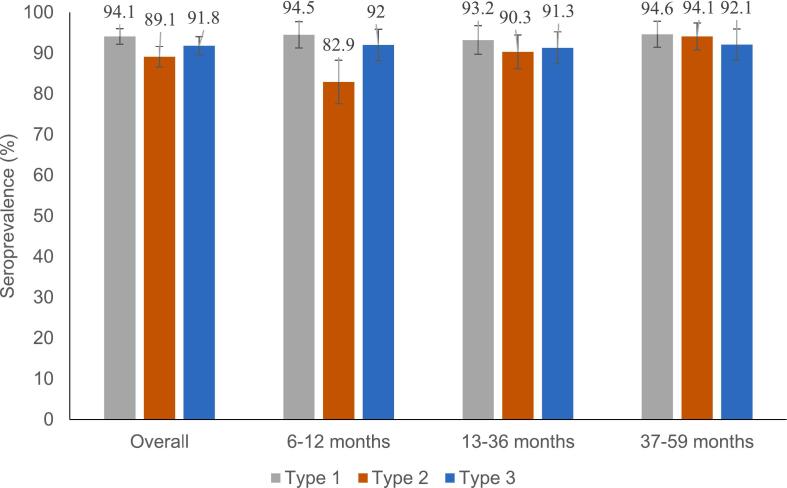
Overall seroprevalence of all three serotypes and by age groups: 6–12 months, 13–36 months and 37–59 months. Abbreviations – type 1, SP1; type 2, SP2; type 3, SP3.

The overall seroprevalence rates for poliovirus type 1, type 2, and type 3 were 94.1 % (95 % CI: 91.9–95.7), 89.1 % (95 % CI: 86.4–95.4), and 91.8 % (95 % CI: 89.3–93.7), respectively. When analyzed by serotype, the seroprevalence rates for poliovirus types 1 and 3 were above 90 % in all three age categories. For type 2, the seroprevalence was lower at 83 % in children between 6 and 12 months of age as compared to children above 1 year who had ≥90 % (*p* = 0.001) (Fig. 3).

**Fig. 4:**
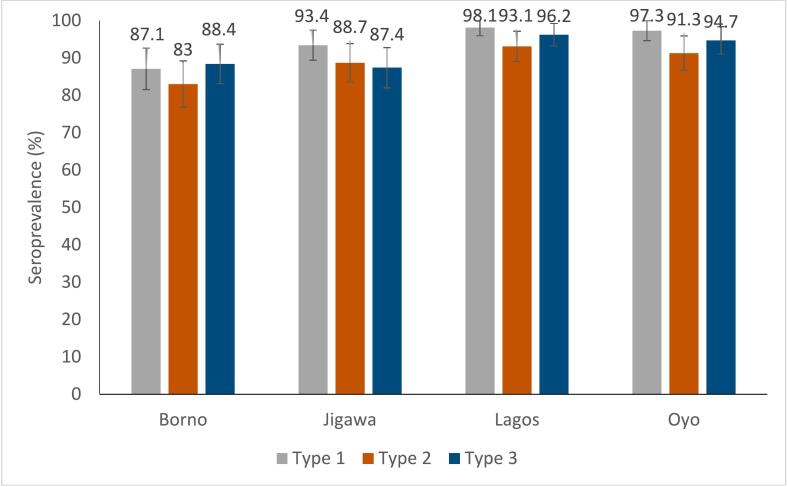
Types 1, 2 and 3 seroprevalences by study site. Abbreviations – serotype 1, SP1; serotype 2, SP2; serotype 3, SP3.

Seroprevalence rates were compared between the states. The seroprevalence for poliovirus type 1 ranged from 87.1 % (95 % CI: 80.8–91.6) in Borno to 98.1 % (95 % CI: 94.6–99.4) in Lagos. Similarly, the seroprevalence for poliovirus type 2 varied from 83 % (95 % CI: 76.1–88.2) in Borno to 93.1 % (95 % CI: 88.0–96.1) in Lagos. Furthermore, the seroprevalence for poliovirus type 3 ranged from 87.4 % (95 % CI: 81.2–91.8) in Jigawa to 96.2 % (95 % CI: 92.0–98.3) in Lagos (Fig. 4).

**Fig. 5:**
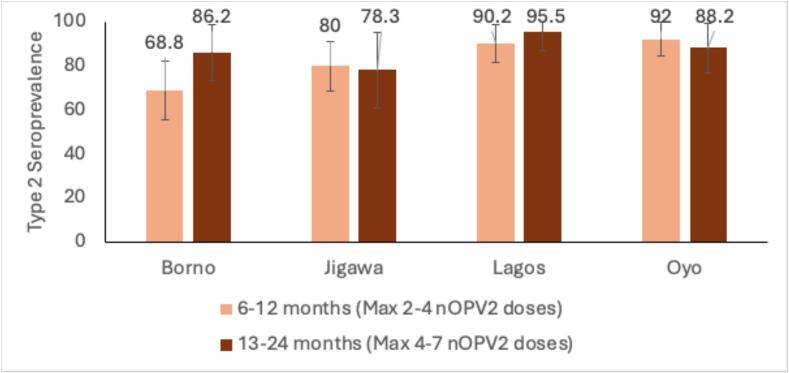
Type 2 seroprevalence by number of nOPV2 outbreak response campaigns among children less than 2 years.

In the northern states of Borno and Jigawa, children aged 6 to 12 months were potentially exposed to 3 to 4 nOPV2 vaccination campaigns, while only 2 campaigns were held in Lagos and Oyo in the year preceding seroprevalence. Despite the higher number of campaigns in the northern states, type 2 seroprevalence was lower in Borno and Jigawa (69 % and 80 %, respectively) compared to Lagos and Oyo (90 % and 92 %, respectively; *p* < 0.001). This disparity between the states was similarly observed in children aged 13–24 months with higher number of doses as well (Fig. 5).

### 3.2 Titer distribution

The overall median titers (Table 2) were high for all three poliovirus serotypes. For types 1 and 3, a declining trend was observed with increasing age while type 2 demonstrated an increasing trend with age which achieved level of significance (*p* = 0.032) between younger and older cohorts.

**Table 2:** Median neutralizing antibody titers with 95 % Bootstrap CIs of all three serotypes by age categories.

Antibody titers	6–12 months		13–36 months		37–59 months	
	Median	95 % CI	Median	95 % CI	Median	95 % CI
Type 1	10.2	10.2–10.5	9.5	8.8–9.8	8.5	8.2–9.0
Type 2	7.8	6.8–8.2	7.7	7.2–8.5	8.5	8.2–8.8
Type 3	9.5	9.3–9.8	8.5	8.2–8.8	7.5	7.2–7.8

**Fig. 6:**
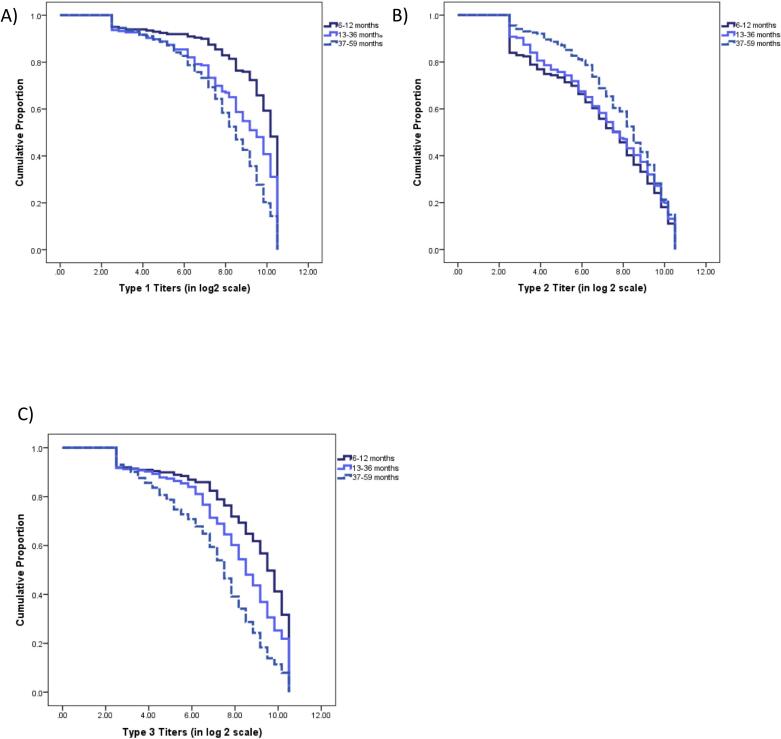
Reverse cumulative neutralizing antibody titer distribution of all three serotypes by age categories. A) Serotype 1, B) Serotype 2, C) Serotype 3.

The reverse cumulative titer distribution (Fig. 6) shows the titer distribution of all three serotypes across the three age sub-categories.

Association of sociodemographic characteristics, nutritional indices, and vaccination history with all three serotypes is also presented here (Table 3**)**. Study states, underweight children, parents' education, total bOPV doses, and receipt of IPV in RI were significantly associated with seroprevalence for 1 and 3 serotypes. Older group children (age > 1 year) had significantly higher rates of type 2 seropositivity (≥90 %) compared to younger children (82.9 %) (*P* = 0.002). Children whose parents had some formal education had significantly higher levels of seropositivity compared to those whose parents had no formal education or never attended school (91 % vs 77 %; *p* = 0.001). Furthermore, children from households with ≤2 children under the age of five years had a significantly higher type 2 seroprevalence compared to households with ≥3 under the age of five years (*p* < 0.001). The proportion of children who were seropositive among those who received IPV in RI was significantly higher as compared to those who did not receive IPV in RI (90 % vs 15 %; *p* = 0.035).

**Table 3:** Association of socio-demographic, nutritional indices and vaccination history with all three serotypes.

Variables	SP1			SP2			SP3		
	%	n	P value	%	n	P value	%	n	P value
Age categories									
6–12 months	94.5	188	0.845	82.9	165	0.002	92.0	183	0.948
13–36 months	93.2	192		90.3	186		91.3	188	
37–59 months	94.6	191		94.1	190		92.1	186	
									
Gender (Female)	94.6	247	0.729	86.6	226	0.088	91.2	238	0.658
Study states									
Borno	87.1	128	<0.001	82.9	122	0.035	88.4	130	0.007
Jigawa	93.4	141		88.7	134		87.4	132	
Lagos	98.1	156		93.1	148		96.2	153	
Oyo	97.3	146		91.3	137		94.7	142	
									
Wasted	93.2	109	0.666	88.0	103	0.623	93.2	109	0.581
									
Underweight	90.1	128	0.041	86.6	123	0.283	88.0	125	0.080
									
Stunted	91.5	139	0.164	86.2	131	0.229	85.5	130	0.003
Mother's education level									
No formal education	77.3	58	<0.001	77.3	58	0.001	77.3	58	<0.001
Some education	96.4	513		90.7	480		93.8	496	
Father's education level									
No formal education	78.7	37	<0.001	74.5	35	0.003	74.5	35	<0.001
Some education	95.3	529		90.3	501		93.2	517	
Number of children < 5 yrs in the household									
1–2	94.3	509	0.575	91.1	492	<0.001	92.8	501	0.014
≥3	92.3	60		72.3	47		83.1	54	
Total bOPV doses									
1–3	79.3	23	0.009				72.4	21	0.002
4–6	95.0	441					93.1	432	
>6	93.9	107					91.2	104	
									
Received mOPV2 in SIA (among children who received mOPV2)				60.5	125	0.880			
nOPV2 doses									
≤1				88.2	231	0.514			
≥2				89.9	310				
									
Received IPV through RI	94.5	446	0.011	90.0	425	0.035	92.6	437	0.012
									
Received IPV through SIA	92.1	35	0.493	92.1	35	0.788	89.5	34	0.539

As the main focus of the survey was to assess type 2 seroprevalence, the adjusted analysis was done for type 2 seroprevalence (Table 4). Since our focus was on nOPV2 impact, we included <2 years age group in the adjusted analysis for studying dose relationship with nOPV2 and excluded mOPV2 from this analysis applicable only to >2 years. <2 years children received only nOPV2 and no mOPV2, while children >2 years had received both nOPV2 and mOPV2 over the years.

**Table 4:** Adjusted analysis for type 2 seroprevalence.

Variables	OR	95 % CI		P value
Age Categories				
6–12 months	0.46	0.25	0.86	0.015
>12 months				
				
Gender - Female	0.67	0.38	1.17	0.163
Study states				
Borno	0.65	0.27	1.54	0.431
Jigawa	1.46	0.54	3.89	0.759
Lagos	1.14	0.45	2.85	0.549
Oyo				
Mother's education level				
No formal education	0.44	0.19	1.01	0.053
Some formal education				
Father's education level				
No formal education	0.68	0.27	1.75	0.431
Some formal education				
Number of children < 5 years in the household				
1–2	0.21	0.10	0.43	<0.001
≥3	1.22	0.57	2.63	
				
IPV (in RI or SIA)	1.22	0.57	2.63	0.597
nOPV2 doses				
≤1				
≥2	0.77	0.39	1.51	0.442

The only characteristics that demonstrated a significant risk of lower type 2 seroprevalence were age of the child and the number of children under 5 years in a household. Younger children (<1 year) were 56 % less likely to become seropositive as compared to older children (*P* value = 0.015). The odds of being seropositive was lower (OR = 0.21; *p* < 0.001) in those households which had 3 or more children under five years of age.

## 4 Discussion

The overall seroprevalence rate for poliovirus types 1, 2, and 3 was ≥89 % across four states in Nigeria. This is attributed to administration of different polio vaccines in RI (bOPV, IPV) as well as IPDs (nOPV2, mOPV2, bOPV, IPV/fIPV). Type 2 seroprevalence was reported at 89 %, and is the combined effect of multiple nOPV2, mOPV2 and IPV/fIPV IPDs in the study areas and IPV through RI. It is difficult to segregate the neutralizing antibodies contributed by nOPV2 or establish any direct dose effect relationship in this complex background of potential exposure to different vaccines and doses. We tried to compare the type 2 seroprevalence obtained in Nigeria with similar studies elsewhere. In Tajikistan, type 2 seroprevalence was 83 % after two nOPV2 campaigns implemented as outbreak response [21]. On the contrary, a study in Liberia showed very low type 2 seroprevalence after two nOPV2 doses at 38 % [22]. The differences in seroprevalence in different settings could be explained by variable field efficacy of OPVs in different populations and the coverage.

The observed seroprevalence of type 2 aligns well with the declining trend of cVDPV2 in Nigeria from 2021 to 2023, subsequent to outbreak response employing multiple nOPV2 campaigns. While the outbreaks stopped after two nOPV2 campaigns in many other countries, type 2 circulation continues in Nigeria. It is possible multiple vaccine doses are needed due to lower vaccine delivery or efficacy, especially in northern states of Nigeria.

From the start of nOPV2 use, between 2021 and 2022 and prior to the seroprevalence sampling, Jigawa and Borno in the north conducted 6–7 rounds with nOPV2, while Lagos and Oyo, administered 4 rounds each. Despite Borno and Jigawa implementing more nOPV2 campaigns, lower type 2 polio seroprevalence rates among children aged 6–12 months and 13–24 month were observed in these states, while states with fewer nOPV2 rounds, such as Lagos and Oyo, exhibited higher seroprevalence rates. The discrepancies in seroprevalence rates of type 2 polio among different states in Nigeria likely highlight gaps in vaccine coverage in different settings [23].

Comparing seroprevalence among different age subgroups within the study population provides valuable programmatic information. Lower type 2 seroprevalence of 82.9 % in the youngest age group of 6–12 months, even lower in Borno and Jigawa states (68 % in Borno and 80 % in Jigawa), leaves a significant immunity gap for type 2. This gap along with the addition of newborn cohorts will continue to sustain a large pool of susceptible individuals at risk for continuing poliovirus transmission. This underscores the need for prioritized attention to newborns and younger cohorts during IPDs by tracking them to ensure they receive every dose in the schedule. Higher seroprevalence with increasing age is likely an impact of additional doses received over time.

The median antibody titers for serotypes 1 and 3 showed a decreasing trend with age, likely attributable to waning immunity over time and/or, a minimal or no exposure to these serotypes beyond infancy, compounded by the absence of significant exposure to bOPV in recent years from SIAs. In contrast, antibody titers for serotype 2 remained stable or increased due to repeated exposures from mOPV2, nOPV2, IPV, and the ongoing transmission of VDPV2 in some of these populations.

IPV plays a crucial role in closing type 2 immunity gaps in those who previously received OPV [24,25]. Introduction of IPV in essential immunization and its coverage is also an important factor for type 2 immunity. A notable association between the administration of IPV as part of essential immunization and increased type 2 seroprevalence was also observed in the unadjusted analysis in our study (Table 3). Overall IPV coverage remains low at 62 % [8] (between 2020 and 2022) in Nigeria and there are significant disparities in coverage between northern and southern regions due to factors like socio-economic status, healthcare infrastructure, cultural beliefs and geographical accessibility [26].

Parental education level was also found to influence type 2 seroprevalence. Children whose parents had formal or higher levels of education, showed significantly higher levels of seropositivity. This finding may reflect better awareness and understanding of the importance of immunization among educated parents and better compliance of essential vaccination [27]. Interestingly, households with more than two children under the age of five had lower type 2 seroprevalence, which may be attributed to challenges in ensuring timely and complete vaccination coverage within larger households. This highlights the need for health education initiatives targeting parents and caregivers, particularly those with lower levels of education and larger households, to provide due attention to every child.

Nutritional indices did not show a significant association with type 2 seroprevalence in this study. However, it is important to note that nutritional status can influence OPV vaccine response and overall immunity in some populations [28,29]. In our study, underweight and stunted children were significantly associated with type 1 and type 3 seroprevalence, respectively, but no such association was found with type 2.

The study had some limitations. The cross-sectional design restricts our ability to establish causal relationships between identified factors and seroprevalence. The reliance on caregiver reports for vaccination history may introduce measurement error due to recall bias despite best efforts made at history taking. Additionally, the study sample was limited to selected health facilities, which may not fully represent the general population in Nigeria.

Future studies should consider longitudinal designs to assess the durability of immunity conferred by nOPV2 and its long-term impact on type 2 poliovirus transmission as suggested in previous models [30]. Larger-scale studies involving diverse populations and geographical areas would provide a more comprehensive understanding of the relationship between nOPV2 and seroprevalence to type 2 polio.

In conclusion, the present study demonstrates high overall seroprevalence rates for poliovirus types 1, 2, and 3 among children in Nigeria, albeit there is variance at the state-, serotype-, and age-levels as discussed. The combined impacts of essential immunization (IPV) and nOPV2 response campaigns appear to have contributed to the overall level of seroprevalence against type 2 polio in the studied population. Age, parental education, household composition, and vaccination history were identified as factors influencing type 2 seroprevalence. These findings support the potential of nOPV2 as a valuable tool in polio eradication efforts and underscore the importance of targeted vaccination strategies, improved vaccine coverage, and regional evidence-based interventions to ensure sustained polio eradication efforts in Nigeria [31,32].

Since at present, use of nOPV2 is restricted to the outbreak response, its introduction in RI might help with higher level type 2 population immunity.

## Disclaimer

The findings in this article are those of the authors and do not necessarily represent the official position of the contributing agencies, including the US Centers for Disease Control and Prevention.

Disclaimer: The designations employed and the presentation of the material in this publication do not imply the expression of any opinion whatsoever on the part of WHO concerning the legal status of any country, territory, city or area or of its authorities, or concerning the delimitation of its frontiers or boundaries. Dotted and dashed lines on maps represent approximate border lines for which there may not yet be full agreement.

## CRediT authorship contribution statement

**Zubairu Iliyasu:** Writing – review & editing, Writing – original draft, Methodology, Investigation, Conceptualization. **Abba Ahmed Danzomo:** Supervision, Project administration, Methodology, Conceptualization. **Visalakshi Jeyaseelan:** Writing – review & editing, Writing – original draft, Validation, Software, Investigation, Formal analysis. **Mustapha Modu Gofama:** Writing – review & editing, Supervision, Methodology, Conceptualization. **Patricia Eyanya Akintan:** Writing – review & editing, Methodology, Investigation. **Olugbenga Oyewumi Akinrinoye:** Writing – review & editing, Methodology, Investigation. **Umar Also:** Writing – review & editing, Supervision, Methodology, Investigation. **Hamisu Abdullahi:** Conceptualization. **Kabir Yusuf Mawashi:** Methodology, Conceptualization. **Auwal Umar Gajida:** Methodology, Investigation, Conceptualization. **Giovanna Sifontes:** Investigation, Methodology, Validation, Writing – review & editing. **Bernardo A. Mainou:** Writing – review & editing, Writing – original draft, Methodology. **Rocio Lopez Cavestany:** Writing – review & editing, Writing – original draft, Conceptualization. **Ondrej Mach:** Writing – review & editing, Writing – original draft, Validation, Methodology, Conceptualization. **Harish Verma:** Writing – review & editing, Writing – original draft, Supervision, Resources, Project administration, Methodology, Investigation, Funding acquisition, Conceptualization.

## Declaration of competing interest

The authors declare that they have no known competing financial interests or personal relationships that could have appeared to influence the work reported in this paper.

## Data Availability

Data will be made available on request.
